# Modulation of the Gut Microbiota by Nutrition and Its Relationship to Epigenetics

**DOI:** 10.3390/ijms25021228

**Published:** 2024-01-19

**Authors:** Katarzyna Ferenc, Aneta Sokal-Dembowska, Kacper Helma, Elżbieta Motyka, Sara Jarmakiewicz-Czaja, Rafał Filip

**Affiliations:** 1 Institute of Medicine, Medical College of Rzeszow University, 35-959 Rzeszow, Poland; 2 Institute of Health Sciences, Medical College of Rzeszow University, 35-959 Rzeszow, Poland; 3 Centre for Innovative Research in Medical and Natural Sciences, Medical College of Rzeszow University, 35-959 Rzeszow, Poland; 4 Department of Gastroenterology with IBD Unit, Clinical Hospital No. 2, 35-301 Rzeszow, Poland

**Keywords:** epigenetics, gut microbiota, nutrients

## Abstract

The intestinal microbiota is a community of microorganisms inhabiting the human intestines, potentially influencing both physiological and pathophysiological processes in the human body. Existing evidence suggests that nutrients can influence the modulation of the gut microbiota. However, there is still limited evidence regarding the effects of vitamin and mineral supplementation on the human gut microbiota through epigenetic modification. It is plausible that maintaining an adequate dietary intake of vitamin D, iron, fibre, zinc and magnesium may have a beneficial effect on alleviating inflammation in the body, reducing oxidative stress, and improving the condition of the intestinal microbiota through various epigenetic mechanisms. Moreover, epigenetics involves alterations in the phenotype of a cell without changing its fundamental DNA sequence. It appears that the modulation of the microbiota by various nutrients may lead to epigenetic regulation. The correlations between microbiota and epigenetics are potentially interdependent. Therefore, the primary objective of this review is to identify the complex relationships between diet, gut microbiota, and epigenetic regulation. These interactions could play a crucial role in systemic health.

## 1. Introduction

In recent years, increasing evidence has suggested a link between intestinal microbiota modulation and epigenetic regulation. The impact of diet on the composition and diversity of the intestinal microbiome has also been documented, which may consequently have long-term effects on overall health [1,2]. Understanding the interactions between nutrition, gut microbiota and epigenetic regulation seems essential in the context of exploring potential therapeutic interventions that aim to improve overall health by modulating the microbiota to favourably impact epigenetic regulation. The aim of this review is to examine how individual nutrients can impact the gut microbiota composition, subsequently affecting gene expression regulation through epigenetic mechanisms.

## 2. Gut Microbiota

The gut microbiota is the totality of microorganisms such as bacteria, fungi, viruses and archaeons that reside in the intestines. The human intestinal microbiome is a complex ecosystem consisting of microorganisms that permanently reside in the body, or autochthonous components, and those that temporarily inhabit the body, or allochthonous components [3]. The human gut is dominated by the *Firmicutes*, *Bacteroidetes*, *Actinobacteria* and *Proteobacteria* phyla [4,5]. There are more than 1000 bacterial species in the gut, which encode about 5 million genes [6]. In some studies, the gut microbiome is repositioned as an “essential organ” of humans [7,8]. Therefore, the gut microbiota can have an impact on many factors related to health or disease.

### The Roles of the Gut Microbiota

The intestinal microbiota has been shown to influence numerous functions in the human body, including being involved in immunomodulation, nutrient metabolism, and intestinal protective functions.

The gut microbiota has been shown to influence the modulation, maturation, and functioning of the immune system. Commensal microorganisms are essential for the differentiation of the immune system cells. Moreover, they can play a role in gut-associated lymphoid tissue (GALT) maturation [9]. An example of immunomodulatory actions is the activation of interleukin 1β (IL1β) through commensal microorganisms via TLR-MyD88 signalling, which, in turn, can activate IL17 [10]. Another example of such actions is the regulation of regulatory T cells by metabolites derived from the intestinal microbiota, such as short-chain fatty acids (SCFAs) and bile acids (BA) [11]. Furthermore, intestinal dysbiosis reduces immune tolerance to the commensal microbiota, which in turn may affect the Treg and Th17 imbalance, as well as the balance of the intestinal barrier [12]. The gut microbiota has also been shown to affect immunoglobulin production [13].

Maintaining normal intestinal barrier function is important to maintain host health. Besides the commensal gut microbiota, the intestinal barrier comprises mucus, protective proteins, intestinal epithelial cells, and immune cells [14]. Microbiota-induced cleavage of meprinβ is required for the secretion of mucus from the small intestine. Furthermore, the composition of the intestinal microbiota also affects the properties of mucus. In addition, bacterial adhesion affects the composition of the intestinal microbiota. Commensal bacteria degrade and utilize mucin glycans as a nutrient, and similar interactions are also observed among pathogenic bacteria [15]. Shi et al. emphasize the key role of the gut microbiota on host mucosal immunity [16].

Another of the functions of the gut microbiota is nutrient metabolism. The gut microbiota exhibits the ability to modify primary BA. Transformation of primary Bas results in the formation of secondary Bas, e.g., ursodeoxycholic acid or deoxycholic acid. Metabolism of the gut microbiota can alter the bioactivity or bioavailability of BA [17]. In addition, BA has also been shown to affect the abundance and composition of the gut microbiota [18]. Bacterial fermentation results in the production of SCFAs. According to Martin-Gallausiaux et al., via the production of SCFAs, the gut microbiota communicates with host cells, modulating selected cellular mechanisms [19]. It also synthesises other bioactive compounds, e.g., B vitamins, vitamin K2, vitamin A [20,21]. This is a two-way relationship, as many of the dietary factors can also affect the composition of the gut microbiota.

The influence of long-term dietary patterns on the incidence of individual enterotypes has been shown to be linked. Wu et al. reported the association of the *Bacteroides* enterotype with a high-protein and high-fat diet and the *Prevotella* enterotype associated with a carbohydrate diet [1]. Many dietary components have been shown to have a modulating effect on the gut microbiota. Some of the more commonly studied components are vitamins A, E, B6, and C. They may exhibit antimicrobial activity, which translates into changes that occur in the intestinal microbiota, in terms of metabolic activity and its composition [2]. Furthermore, a high-fibre diet shows beneficial effects on host health, as anaerobic bacteria provide the aforementioned SCFAs via carbohydrate fermentation [22]. Bioactive dietary components such as ferulic acids, caffeic hydroxycinnamates, and coumaric also have a beneficial effect on the quality of the gut microbiota. *Lactobacillus* and *Bifidobacterium* show the ability to release these components from the conjugated plant form [23]. On the other hand, adverse effects on the intestinal microbiota are observed following a Western-type diet, characterized by high consumption of highly processed foods, high amounts of saturated fatty acids and trans fatty acids, high salt content, and low dietary fibre. It can lead to intestinal dysbiosis and low-grade inflammation, leading to intestinal barrier dysfunction [24]. In addition, selected food additives, e.g., carboxymethylcellulose and polysorbate 80, can also adversely affect the gut microbiota [25,26]. In addition to an inadequate diet, a sedentary lifestyle is detrimental to the gut microbiota.

**Figure 1:**
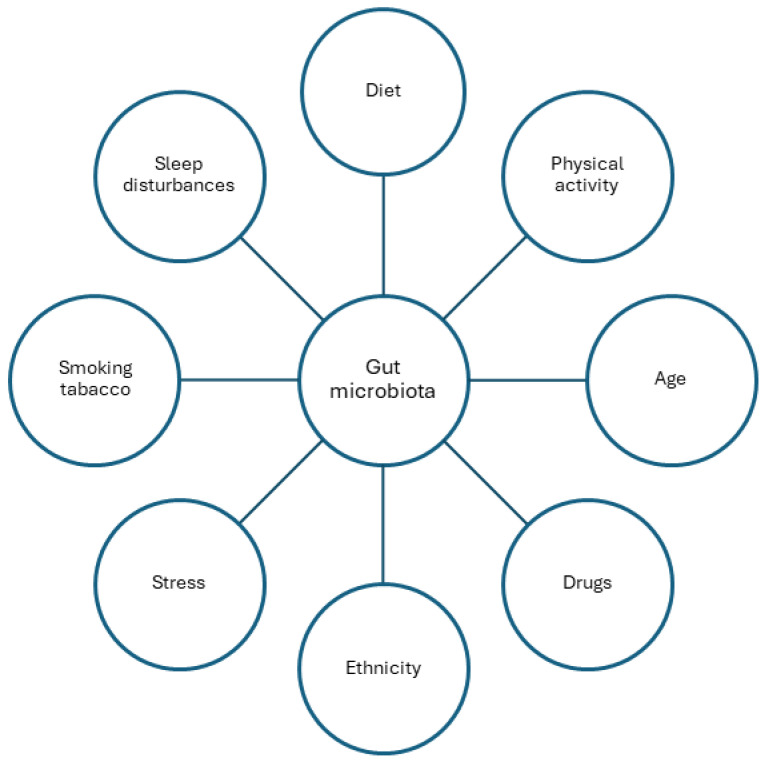
Factors affecting the composition of the gut microbiota.

Another factor affecting the type and quantity of gut microbiota is physical activity. Variations have been observed in physically active people and those leading sedentary lifestyles. The type of exercise undertaken and the level of exercise may also correlate with changes in the gut microbiota [27,28,29,30,31,32,33,34]. Numerous studies have indicated that the composition of the gut microbiota undergoes changes with age [35,36,37,38,39]. Alterations in both the composition and quantity of the gut microbiota have also been observed with pharmacotherapy, including the use of proton pump inhibitors [40]. The use of drugs can impact the gut microbiota and, in turn, the gut microbiota can influence drug metabolism [41,42,43]. Ethnicity and place of residence have also been identified as influencing factors [44,45,46,47,48,49,50,51]. In addition, other factors such as stress, tobacco smoking and sleep disorders play a role in shaping the gut microbiota [52,53,54,55,56,57,58,59,60,61,62] (Figure 1).

## 3. Epigenetic Regulation

Epigenetics includes any mechanism that can ultimately cause heritable changes in a cell’s phenotype without altering the underlying DNA sequence [63]. Epigenetic mechanisms are involved in regulating and overseeing a wide variety of physiological and pathophysiological processes [64]. Proper epigenetic processes are very important for cell growth and proliferation and for transcriptional regulation and genome integrity [65]. There are three main epigenetic mechanisms. These include DNA methylation, histone modification, and RNA interference [66]. It now appears that epigenetic changes can be induced by the environment. Importantly, the consequences of these mechanisms can be reversible [67].

### 3.1. Epigenetic Mechanism

DNA methylation is one of the most important epigenetic mechanisms [68]. There are a number of possible DNA methylations, such as 4-methylcytosine (4 mC), 5-methylcytosine (5 mC), and N6-methyladenine (6 mA) [69]. 6 mA and 4 mC are found in the prokaryotic genome. In contrast, 5 mC is characteristic of eukaryotes. In addition, 5 mC is among the most studied processes of DNA methylation [70]. This is a process involving the transfer of a methyl group to the C5 position of a cytosine in a di-nucleotide (CpG sequence). This is done by the enzymatic action of a group of DNA methyltransferases (DNMTs) [69]. Large numbers of CpG sequences can be found in certain regions of DNA. They are called CpG islands [71]. DNA hypermethylation of specific genes and general methylation changes are often linked to the induction of many disease processes [72]. DNA methylation is a process under dynamic control. It can be reversed by cell division when methyl groups are lost or during demethylation [73].

Histone modifications are important epigenetic processes that are responsible for regulating basic biochemical processes. This regulation results from chromatin modifications and gene expression [74]. Histone modifications can result from processes such as methylation, acetylation, phosphorylation, ubiquitination, and adenosine 5′-diphosphate (ADP) ribosylation [75,76]. Acetylation of histone tails using histone acyltransferases (HATs) most often results in the release of chromatin, thus enabling gene transcription. In contrast, deacetylation using histone deacetylases (HDACs) has the effect of strengthening the bonds between histones and DNA, thereby preventing gene transcription [77].

Non-coding RNA (ncRNA) is a molecule transcribed from DNA which, in contrast, is non-translated into protein [78]. The most well-known ncRNAs are micro RNAs (miRNAs). These are short, single-stranded, 19–24-nucleotide ncRNAs. MiRNAs are responsible for regulating the silencing of protein-coding genes [79]. There are also long ncRNAs that have more than 200 nucleotides. They exist as chromatin remodellers, transcriptional regulators and posttranscriptional regulators [80]. This modification, unlike the previous ones, regulates gene expression at the post-transcriptional level [68].

### 3.2. Epigenetic Regulation and the Gut Microbiota

In recent years, it has been shown that abnormalities in the composition and abundance of the gut microbiota are associated with chronic diseases which include inflammatory bowel disease (IBD), metabolic disorders, or cancer [81]. Intestinal epithelial cells interact with commensal microorganisms. They share the intestinal microbiota with host cells, making them adapted to respond quickly to any bacteria present and their metabolites [82]. In addition, the gut microbiota not only affects the intestinal epithelium, but also the body systemically through the transport of its metabolites [81]. It seems that the microbiota, as a source of external, environmental factors, can affect host cells through which it influences their physiology. This is understood through the issue of epigenetic regulation of the microbiota. The gut microbiota can be a source of a number of important components that can serve as epigenetic cofactors, substrates or regressors of epigenetic enzyme activity [83,84]. In early life, the development of the gut microbiota influences the development of intestinal epithelial cells by modifying epigenetic regulation. Cortese et al. showed that *Lactobacillus acidophilus* and *Bifidobacterium infantis* affected changes in DNA methylation in immature intestinal epithelial cells [85]. Ryan et al. showed that DNA methylation in IBD patients correlated with the composition of the microbiota and with inflammation [86]. In contrast, Tahara et al. showed that *Fusobacterium* in UC patients was associated with increased DNA methylation in genes associated with colon tumourigenesis [87]. Similarly, Sobhani et al. in their study showed that the gut microbiota associated with colorectal cancer also correlated with DNA methylation [88]. Also, similar claims have been made in studies of epigenetic regulation in obese individuals. It has been shown that DNA methylation can affect the gut microbiota in overweight individuals [89]. In addition, it has been shown that acetate and propionate can decrease the activity of HDAC2 and HDAC3. Butyrate, on the other hand, has the effect of inhibiting HDAC1 and HDAC2 activity [90]. On the other hand, miRNAs appear to be involved in the modulation of the microbiota. MiRNAs can regulate the transcription of microbial genes. As a result, they affect the structure of the gut microbiota [91]. Expression of miR-21-5p in intestinal epithelial cells may cause changes in intestinal permeability [92].

## 4. The Interaction between Gut Microbiome and Nutrients through Epigenetic Mechanisms

### 4.1. Vitamin D

Vitamin D is a fat-soluble vitamin synthesized endogenously when exposed to UV sunlight and can be obtained from food sources [93,94]. Vitamin D requirements range from 10–20 mcg depending on age and physiological status. According to the recommendations of the National Institutes of Health (NIH), the recommended dietary allowance (RDA) for adult men and women is 15 mcg (600 IU)/daily, and after the age of 70, the requirement increases to 20 mcg (800 IU)/daily. The upper daily intake (ULs) of vitamin D obtained from dietary sources is considered to be 100 mcg (4000 IU) (>9 years old) [95]. Dietary sources of vitamin D include foods of animal and plant origin and fortified products. Fortified foods may contain forms of D_3_, D_2,_ or the vitamin D metabolite 25-hydroxyvitamin D [96].

#### 4.1.1. Vitamin D/Vitamin D Receptor (VDR) Axis

The vitamin D/vitamin D receptor (VDR) axis is critical to maintaining human health. Vitamin D can perform its biological functions by binding through the VDR. It is believed that vitamin D may play an important role in the occurrence and development of autoimmune diseases, and the presence of specific alleles and genotypes of a single VDR nucleotide modulates their development. Moreover, the vitamin D/VDR axis regulates the expression of genes involved in various functions [97], including:

- cell growth and differentiation [98]
- regulation of calcium and phosphate metabolism—differentiation of osteoclasts, mineralization of the bone matrix [98,99]
- autocrine and paracrine function [99]
- immunomodulatory function—VDR is expressed in immune cells, including activated or naive CD4+ and CD8+ T lymphocytes, B lymphocytes, neutrophils and antigen-presenting cells: dendritic cells, monocytes, macrophages [98]
- endocrine function [94]

#### 4.1.2. Microbiota, Epigenetics and Vitamin D

The vitamin D/VDR axis is largely influenced by environmental factors such as diet and sun exposure [97]. In addition to the previously mentioned important role of VDR in immune response, it is also responsible for regulating the inflammatory response and maintaining intestinal homeostasis [98]. *VDR* was considered the first gene shaping the human microbiome [100] and is additionally involved in the epigenetic modulation of the host [97]. VDR signalling is crucial in the protection of the gastrointestinal epithelium, and vitamin D deficiency may cause increased intestinal permeability and bacterial translocation [98]. Anderson et al. showed that vitamin D3 supplementation during pregnancy from the end of the second trimester at a dose of 3800 UI until the 4th–6th week after delivery may be a potential factor differentiating DNA methylation in the epigenomes of the mother and infant. Vitamin D supplementation was associated with an increase in maternal leukocyte methylation in genes regulating, among others, vascular/endothelial development and immune function [101]. VDR regulates the expression of the peptides claudin 2, cathelicidin and β-defensins, which have a barrier function and prevent contact of intestinal microorganisms with the epithelial surface [102]. Vitamin D, through nuclear interactions with the VDR, may influence bacterial colonization, and its deficiency may lead to disturbances in intestinal homeostasis. However, the introduction of supplementation may have a positive impact on the composition of the host’s microbiota [94]. There is likely a positive relationship between VDR signalling and butyrate through its effect on VDR protein expression [103].

Proper VDR function can affect several genes related to inflammation, autophagy or barrier function. VDR is associated with the regulation of more than 600 genes. D/VDR can be regulated via the expression of CYP27B1. Increased CYP27B1 expression and decreased VDR levels are observed in colitis, resulting in reduced inflammation and improved VDR signalling. VDR can bind to histones, thereby inhibiting transcription of ZO-1, occludin, and claudin-5 genes. In addition, VDR increases tight junction protein -*cluadin-2*, and increased inflammatory responses in the intestine are closely associated with overactivity of cluadin-2. Improved VDR signalling and reduced inflammation are also observed as a result of increased expression of local CYP28B1 [100].

VDR is involved in the differentiation of regulatory T cells and Paneth cells and the release of antimicrobial peptides [100]. Studies in animal models have shown that VDR knockout mice exhibit defective Paneth cell function leading to impaired antimicrobial activity and increased inflammatory responses [104]. In addition, reduced levels of VDR or lack of the active form of vitamin D have been linked to an increase in *Proteobacteria* and *Bacteriodetes* with a concomitant decrease in the number of *Lactobacillus* in the intestinal microbiota and impaired intestinal barrier function [100,105]. In a study by Singh et al., 12 weeks of vitamin D supplementation in healthy women with vitamin D deficiency at a dose of 50,000 UI/week led to a higher *Bacterioidetes*/*Firmicutes* ratio and an increase in the number of *Actinobacteria* and *Verrucomicrobia* phyla [106]. In a randomized, double-blind placebo-controlled study by Naderpoor et al., changes in the faecal microflora were observed in overweight or patients suffering from obesity. The subjects received a saturating dose of cholecalciferol of 100,000 IU followed by 4000 IU/day for 16 weeks or a placebo. Vitamin D supplementation was found to be associated with higher abundance of the genus *Lachnospira* and lower abundance of the genus *Blautia*. Additionally, 25(OH)D concentrations above 75 nmol/L were associated with higher abundance of the genus *Coprococcus* and lower abundance of the genus *Ruminococcus* compared to those with 25(OH)D concentrations below 50 nmol/L. The authors suggest that compensating for vitamin D deficiency may positively affect BMI and insulin resistance and mitigate inflammation in this group [107]. On the other hand, the use of probiotic therapy may contribute to an increase in circulating vitamin D, thereby influencing a reduction in inflammation and improving VDR signalling [100].

MiRNAs as a class of small non-coding RNAs are also currently being targeted for research into their association with the VDR, the development of inflammation, and intestinal fibrosis. However, there are still not many researchers who have succeeded in finding links between miRNAs and VDR functions [100]. Intestinal epithelial VDR levels are down-regulated via a mechanism mediated by miRNAs. Some data indicate that both Mir-25B and Mir-346 target the VDR [108].

The VDR can regulate autophagy and apoptosis. Among other things, this mechanism depends on the action of vitamin D in ATG16L1, and increased apoptosis is a major cause of increased mucosal permeability [100,109]. Inhibition of inflammation-induced intestinal epithelial cell apoptosis can be inhibited by VDR signalling, such that it maintains the integrity of the mucosal barrier [109].

Research on dysbiosis and VDR, especially in the course of various disease states, is still limited. Nonetheless, improving nutritional status may be one way to restore gastrointestinal homeostasis, maintain the intestinal barrier, and preserve the proper ratio of microorganisms to each other. However, further studies are needed to investigate the role of vitamin D/VDR in the modulation of intestinal microflora and anti-inflammatory effects through epigenetic changes.

### 4.2. Iron

Iron requirements vary depending on age, gender, physiological status, and diet (vegetarians should increase the intake of this element in their diet by 1.8 times). The average daily intake of iron in the human diet is 7 to 15 mg, as only 1 to 2 mg is absorbed by the intestinal tract [108,110]. According the NIH recommendations, the daily intake for adult men and women, is 8 mg and 18 mg, respectively. In addition, iron requirements increase during lactation to 27 mg. A dose of 45 mg/day is considered the upper daily intake, although in iron-deficient states of the body, in patients diagnosed with anaemia, the doctor may order a dose higher than the ULs [111]. Iron can be supplied with food or dietary supplements, and is absorbed mainly in the duodenum and proximal part of the jejunum. In food, iron occurs in haeme and non-haeme forms.

The presence of calcium, phytic acid, or polyphenols affect the absorption and bioavailability of iron in the gastrointestinal tract [108]. Therefore, it is important to be able to properly prepare and compose meals to increase the absorption of this element. Improved iron absorption can be achieved by adding vitamin C to iron-rich meals, while phytic acid reduction can be achieved via soaking, milling, fermenting, or sprouting, among other means [111]. Non-haeme iron is reduced from Fe^3+^ to Fe^2+^ by duodenal cytochrome B on the apical surface of the enterocyte and captured by divalent metal transporter 1 [112]. Iron bioavailability is also influenced by iron regulatory proteins including hepcidin, transferrin, ferritin, ferroportin, iron-responsive element/iron regulatory protein and their corresponding genes [113].

Iron, as a key trace element in the diet, performs a number of important functions in the body, such as:

- responsibility for the transport of oxygen and electrons,
- regulation of gene expression,
- participation in cell differentiation and division,
- aiding in the synthesis of proteins such as mitochondrial aconitase, ribonucleotide reductase, myoglobin and cytochrome proteins.

Both excess and deficiency of iron in the body can be associated with the development of pathological conditions; therefore, its levels are controlled via a number of complex mechanisms. At low iron concentrations, impaired action of antioxidant compounds is observed [108]. However, iron overload can be toxic and may be associated with altered immune responses and organ dysfunction [114,115]. The hormone hepcidin is responsible for maintaining systemic iron homeostasis in the body. Nonetheless, the regulatory process can be disrupted by the development of infections and inflammatory diseases. Increased expression of hepcidin is then observed due to the action of the transcription factor STAT3, LPS, TNF-α, and IL-6, which is a key activator of hepcidin, and probably IL-22 (independent of IL-6) [114]. According to current data, DNA methylation and other epigenetic changes may also influence the maintenance of iron homeostasis in the body [113].

#### Microbiota, Epigenetics, and Iron

The effect of iron on the intestinal epigenome is not yet well understood [116]. In addition to the aforementioned functions of iron in the body, several lines of evidence also point to its effect on the gut microbiome, and its presence is essential for replication and survival of most bacteria [115]. An in vitro study by Celis et al. showed that iron deficiency generally affects the diversity and inhibition of growth of iron-sensitive species. More resistant species occupied niches made available by less resistant ones [117]. Iron deficiency can lead to defective T cell proliferative responses and impaired cytokine production by lymphocytes; on the other hand, it has been shown that excess iron can enhance oxidative and nitrosative stress [118]. In a study by Zimmermann et al., iron supplementation introduced in children suffering from anaemia resulted in an unfavourable ratio of enterobacteriaceae (an increase in bacterial *Enterobacteriaceae* family in faeces) to bifidobacteria and lactobacilli and calprotectin levels. These changes were closely associated with the presence of intestinal inflammation [119]. Dostal et al. showed that low iron concentrations in faecal microflora studies positively correlated with decreases in butyrate and propionate, which consequently may weaken the intestinal microflora barrier, and the presence of SCFAs may be particularly important in the epigenetic regulation of inflammatory responses [118,120].

**Figure 2:**
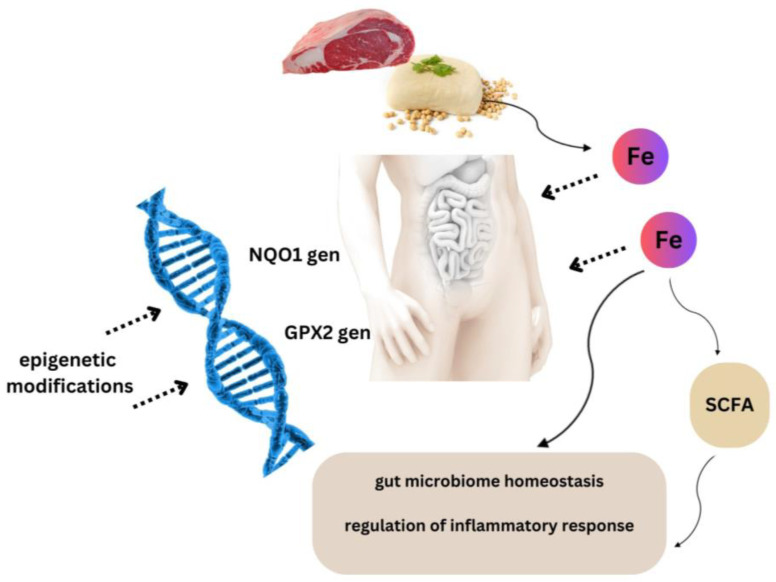
Iron-mediated epigenetic modifications. It is likely that an adequate supply of iron in the diet may be important in epigenetic regulation of inflammatory reactions and maintaining the homeostasis of intestinal microbiota.

The first findings related to iron-dependent epigenetic changes by which cells sense increasing oxidative stress due to excess dietary iron were reported in the work of Harniblow et al. It is likely that chronic iron exposure modifies the epigenetic signatures of colonocytes in vitro and the intestinal mucosa of mice fed a high-iron diet. The authors indicate that chronic iron exposure is required to observe significant changes in hypomethylation. The analysis revealed a significant epigenetic effect on targets of the NRF2 (nuclear erythroid factor 2-related factor 2) pathway. In addition, significant correlations were noted between NQO1 (NAD(P)H Quinone Dehydrogenase) and GPX2 (Glutathione peroxidase 2) demethylation and iron levels in human intestinal tissue. It is likely that iron-mediated epigenetic modifications occur in iron-replete enterocytes (Figure 2) [116].

Understanding the impact of iron on changes in the intestinal microflora through epigenetic changes needs to be studied in depth, especially in inflammatory diseases such as inflammatory bowel disease, since patients are at risk of deficiency of this element and, on the other hand, excess iron may be associated with exacerbation of inflammation.

### 4.3. Short-Chain Fatty Acids

Short-chain fatty acids, such as acetate (C2), propionate (C3), or butyrate (C4), are organic acids that contain 1–6 carbon atoms [121]. They are metabolites formed as a result of the fermentation of dietary fibre by anaerobic intestinal bacteria in the large intestine, where they are mostly absorbed by intestinal epithelial cells [122]. Food substrates for SCFA production include resistant starch, cellulose, inulin, fructo-oligosaccharides, or β-glucans [123]. SCFAs can undergo β-oxidation in mitochondria, eventually leading to ATP production. In the liver, they serve as a source of energy for hepatocytes. SCFAs improve the functioning of the gastrointestinal tract by supporting the integrity of the intestinal barrier and affecting mucus production in the digestive tract, as well as potentially regulating gastrointestinal motility [123].

#### Microbiota, Epigenetics, and SCFAs

Studies indicate that in faecal SCFA production, mostly acetate is formed, followed by propionate and butyrate at a molar ratio of 60:25:15, respectively. This proportion may vary in different segments of the intestine [112]. It has been shown that the quantity and type of SCFAs synthesized may vary depending on the diet. In De Fillips et al., a diet rich in plant-based foods was associated with higher production of SCFAs and provided a more favourable profile of the intestinal microflora [124]. This effect is related to a greater supply of dietary fibre.

**Figure 3:**
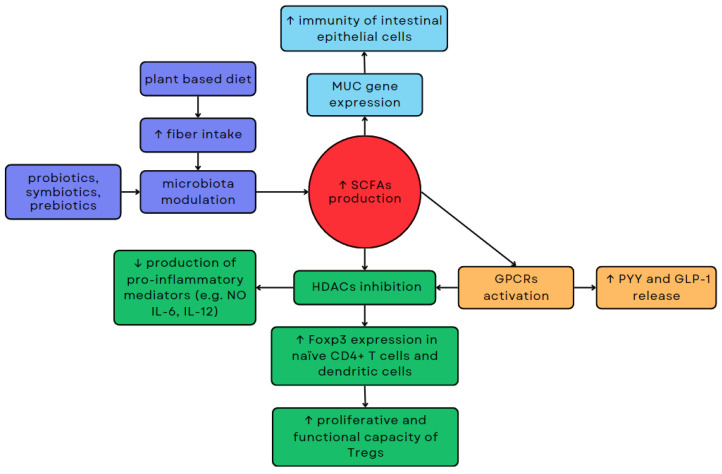
Relationships between microbiota modulation, SCFA production, and epigenetic regulation. Modulation of the microbiota via dietary changes or probiotic use can impact the production of SCFAs, consequently affecting host health through epigenetic mechanisms.

The proportions of SCFAs in the intestine may vary depending on the composition of the gut microflora. It has been demonstrated that bacteria such as *Eubacterium hallii* and *Anaerostipes* spp. can convert acetate and lactate to butyrate [72]. On the other hand, increasing the number of *Acetobacterium*, *Acetogenium*, and *Clostridium* may result in increased production of acetate from butyrate [125]. Acetate can be produced by various intestinal bacteria from pyruvate, but also from H_2_ and CO_2_ by acetogens such as *Blautia hydrogenotrophica*, or from formic acid via the Wood–Ljungdahl pathway [126]. Propionate is primarily synthesized through the succinate pathway involving *Bacteroidetes* and some *Firmicutes* belonging to the *Negativicutes* class. However, it can also be produced through the acrylate and propanediol pathways involving *Proteobacteria*, as well as certain *Firmicutes* like *Roseburia inulinivorans* and *Ruminococcus obeum* [126,127,128]. The proportion of propionate to other major SCFAs in faeces has been shown to increase with the number of *Bacteroidetes* [129]. Butyrate can be produced by *Firmicutes* such as *Faecalibacterium prausnitzii*, *Clostridium leptum*, *Eubacterium rectale* and *Roseburia* spp. [126,127]. Altering the composition of the intestinal microbiota through the use of probiotics, prebiotics, or symbiotics can influence the production of SCFAs, which in turn can impact the health of the host through epigenetic mechanisms (Figure 3) [124].

SCFAs demonstrate the ability to inhibit the activity of HDACs, enzymes that affect gene expression, by increasing histone acetylation [130]. Butyrate is the most potent inhibitor of HDACs, but propionate and acetate also exhibit such effects. In addition to the type of SCFAs, inhibition of HDACs is also influenced by the type of tissue or cell they target [130]. Inhibition of HDACs by SCFAs may affect lymphocytes. Inhibition of HDAC9 by butyrate increases the expression of forkhead box P3 (Foxp3) in naïve CD4+ T cells and dendritic cells, which consequently increases the proliferative and functional capacity of regulatory T cells (Tregs) [130,131]. SCFAs also exhibit anti-inflammatory effects. Inhibition of HDACs by high amounts of butyrate has been shown to reduce the production of pro-inflammatory mediators such as NO, IL-6, and IL-12 [132]. This mechanism may play a role in maintaining homeostasis via the intestinal immune system. Acetate also exhibits anti-inflammatory properties. In vitro, it reduces HDACs activity of human macrophages, which correlates with decreased production of IL-6, IL-8, and TNF-α [130]. Butyrate may increase MUC gene expression, which modulates the synthesis and release of mucin in intestinal epithelial goblet cells, which may consequently improve the immunity of intestinal epithelial cells [133].

SCFAs may also indirectly inhibit HDACs by activating G protein-coupled receptors (GPCRs), such as GPR43, GPR41, and GPR109-A. GPR43 is encoded by the *FFAR2 gene* and has the highest affinity for propionate and acetate and lower affinity for butyrate. The expression of GPR43 occurs throughout the gastrointestinal tract along with cells of the immune and nervous systems. GPR43 may be involved in the differentiation of immune cells. It is expressed on eosinophils, basophils, neutrophils, monocytes, dendritic cells, and mucosal mast cells in bone marrow and the spleen [134]. GPR41, encoded by the *FFAR3 gene*. GPR41 is primarily activated by propionate and butyrate, with a lesser response to acetate. SCFAs can increase the expression of GRP43 and GPR41, subsequently impacting L-cells in the small intestine and colon, thereby enhancing the release of peptides such as intestinal peptide YY (PYY) and glucagon-like peptide 1 (GLP-1) [135]. This mechanism may indicate a potential effect of SCFAs on modulation of insulin release and on feelings of hunger. Administration of 10 g of inulin-propionate ester for 24 weeks has been shown to significantly reduce weight gain in adults with excess body weight [136]. GPR109-A, encoded by the *HCAR2 gene*, is primarily activated by niacin and β-hydroxybutyrate, and to a lesser extent by SCFAs. [134] GPR109-A may be expressed in adipocytes, skin dendritic cells, monocytes, macrophages, and neutrophils [137].

### 4.4. Magnesium

Magnesium (Mg^2+^) is a chemical element commonly found in all living organisms. It is the fourth most abundant mineral in the human body and the second most abundant cation found intracellularly, next to potassium [138]. It is a mineral necessary for the proper functioning and development of the body. It is found in almost all tissues and affects the proper functioning of various systems, such as the cardiovascular, skeletal, muscular, nervous, endocrine, and other systems. It is estimated that magnesium serves as a cofactor for approximately 600 enzymes, and for about 200 enzymes, it acts as an activator [139]. The most important physiological functions of magnesium include its participation in protein biosynthesis; DNA and RNA metabolism; ATP metabolism and all ATP-dependent metabolic reactions; thermoregulation; carbohydrate, lipid and protein breakdown; and insulin metabolism [140]. It also plays an important role in regulating blood pressure and heart muscle function [141,142,143,144]. The absorption of magnesium ions in the gastrointestinal tract is affected by various factors, including dose, age, medical conditions, and alcohol consumption, but also the presence of certain substances in the diet. These substances can inhibit or increase its uptake; for example, cellulose, phytates, and oxalates impair the absorption of magnesium ions, while casein, resistant starch, inulin, and vitamin B6 increase its uptake [140,145]. Many studies show a correlation between chronic magnesium deficiency in the body and a magnesium-deficient diet and an increased risk of diseases such as hypertension, postmenopausal osteoporosis, insulin resistance, type 2 diabetes, migraine pain, depression, colon cancer, and others [146,147,148,149,150,151,152,153]. Plant and animal foods contain magnesium, and it can also be found in mineral water. Foods that are rich sources of magnesium include pumpkin seeds, nuts, cereal grains, bran, groats, brown rice, legumes, spinach, cocoa, bananas, milk and its products, beef, fish, and poultry [140,154,155].

#### Microbiota, Epigenetics, and Magnesium

There are in vivo studies in animal models that provide evidence to support the relationship between magnesium and gut microbiota. Pachikian et al. showed that magnesium-deficient mice had changes in the composition of the intestinal microbiota, specifically a decrease in *Bifidobacterium*, leading to increased intestinal permeability and associated with systemic inflammation and intestinal inflammation [156]. Winther et al. reported that a 6-week magnesium-deficient diet altered the composition of the gut microbiota and further contributed to the development of depressive behaviour in mice [157]. In contrast, another study found that magnesium supplementation in mice suffering from colitis increased levels of *Bifidobacterium*, which improve intestinal health and metabolic balance, while it decreased levels of pro-inflammatory *Enterobacteriaceae*. Magnesium supplementation has been shown to affect the composition, function, and interactions of the gut microbiota in mice. *Enterobacteriaceae* are linked to dysbiosis in inflammatory bowel disease (IBD) in humans, contributing to nutritional deficiencies. Researchers suggest that magnesium supplementation may be a good alternative in alleviating the symptoms of the disease and restoring intestinal microbial balance [158]. However, it is worth noting that García-Legorreta et al., in experiments conducted on magnesium-supplemented rats, showed that in the absence of magnesium deficiency, magnesium supplementation can result in the development of intestinal dysbiosis. Conversely, lower dietary magnesium intake is associated with a greater ability of the microflora to obtain energy from food. The researchers point out that the concentration of minerals in the host must be at the appropriate level for optimal functioning, as there are pathogenic bacteria that can exploit imbalances in mineral levels for their vital functions [159]. Crowley et al., in their study, showed that a functional food ingredient, marine multi-mineral blend (MMB), rich in bioactive magnesium, delivered via diet in rats, significantly increased the microbial diversity and composition of SCFAs in their intestines [160]. Maintaining the proper composition of the intestinal microbiota ecosystem is crucial, leading to the search for tools to maintain the balance of the intestinal gut microbiota, particularly in treating antibiotic-induced dysbiosis [161]. Studies suggest that preparations such as postbiotics or probiotics, when additionally enriched with minerals, work more effectively, enhancing efficacy in positively modulating the gut microbiota and enhancing the bioavailability of minerals [162]. For example, probiotics containing *Lactobacillus* spp. have been shown to significantly increase the bioavailability of magnesium after the consumption of cheese and plant milk [162,163]. Dutch-type ripened cheeses containing probiotic cultures of *Lactobacillus* spp. increase the availability of minerals such as magnesium, calcium, and phosphorus compared to cheeses without added probiotic cultures [164]. Increased bioavailability of magnesium was also observed in goat milk fermented with *Lactobacillus plantarum* [165]. There is little clinical research or evidence of a link between the human gut microbiota and magnesium. There are reports that suggest magnesium supplementation may have a positive effect in obese people with type 2 diabetes and metabolic syndrome and thus magnesium deficiency. However, it is difficult to determine whether the beneficial effect of magnesium has a direct effect on metabolic pathways, an indirect effect on inflammation, or both [166]. Children suffering from chronic constipation were given a combination of the probiotic *Lactobacillus reuteri* and the laxative drug magnesium oxide (MgO). The treatment had the desired effect. MgO combined with the probiotic did not adversely affect the composition of the intestinal microbiota [167]. Fan et al. conducted a study involving 240 participants and showed that magnesium supplementation increased the production of medium-chain fatty acids (MCFA) by intestinal bacteria, which consequently increased plasma MCFA levels [168].

The effect of magnesium on the gut microbiota is an area of intense research, but many questions remain unanswered. Further research is needed to better understand the mechanisms of these interactions and to determine how magnesium supplementation can be used in the context of improving the health of the gut microbiota, and through this, the whole body in humans.

Magnesium absorption occurs in two stages: passive transport, based on electrochemical gradient phenomena; and diffusion, facilitated by the carrier protein transient receptor potential melastatin (TRPM), specifically TRPM6 and TRPM7. TRPM6 is expressed mainly in the intestines, while TRPM7 is ubiquitously expressed in tissues. TRPM6 protein has been shown to be responsible for regulating homeostasis throughout the body, while TRPM7 can regulate magnesium content in the cell [169]. Magnesium is essential for DNA construction, repair, and duplication. It stabilizes the structure of DNA but can also distort the double helix, leading to cell apoptosis, and it can affect cell proliferation [140]. It is hard to talk about the epigenetic role of magnesium in the context of the intestines/gut microbiota itself, although there is no doubt that magnesium as a mineral is included among bioactive compounds which are widely known to participate in epigenetic mechanisms. Bioactive compounds also include probiotics, prebiotics, and postbiotics [170]. We already know that magnesium interacts with the intestinal microbiota (the bacteria of the intestinal microbiota use magnesium for DNA replication, cellular respiration, and their metabolic processes), but it is the intestinal microbiota that directly or indirectly participates in epigenetic mechanisms, through which it affects the health of the gut and the whole body. Magnesium influences the regulation of the number of bacteria and thus the level of SCFAs in the gut as described above. SCFAs have been shown to have a protective effect on DNA transcription, influence the regulation of oncogenes, and exhibit protective potential towards the intestinal barrier to support the treatment of colon cancer or prevent its development [162]. SCFAs can regulate not only peripheral homeostasis, but also mucosal homeostasis. Manipulation in the composition of the gut microbiota, and thus SCFA levels, may become a promising tool in the treatment of dysbiosis, inflammatory diseases such as chronic obstructive pulmonary disease (COPD), asthma, and even allergies [171,172]. For a summary of the above section, see Table 1.

### 4.5. Zinc

Zinc (Zn) is a micronutrient, a trace element essential for the optimal functioning of the human body. It has catalytic, structural, regulatory, and antioxidant functions. It plays an important role in cell metabolism and the stabilization of cell membranes. It is part of more than 300 enzymes. It participates in protein and DNA synthesis; carbohydrate, fat, and protein metabolism; and energy metabolism. It determines the normal function of the immune, nervous, and endocrine systems [173,174]. It influences the process of regeneration and wound healing as well as the perception of taste and smell [175]. Zinc is essential for normal growth and development and the reproductive process. Zinc absorption takes place mainly in the small intestine, and to a lesser extent in the large intestine and stomach [176]. Zinc is found in both animal and plant foods. Foods that are rich sources of zinc include oysters, meat, rennet cheeses, whole grain cereal products, nuts, and seeds [177,178].

#### Microbiota, Epigenetics, and Zinc

Maintaining adequate levels of zinc in the body can contribute to maintaining the balance of the intestinal microbiota, supporting gut health and, through this, the health of the entire body. Zinc is an essential micronutrient for the bacteria of the intestinal microbiota. About 20% of the zinc provided from food is utilized by gut bacteria. Studies indicate that zinc deficiency alters the structure and composition of the gut microbiota, resulting in decreased biodiversity, increased inflammatory markers, and impaired functional potential related to gut–brain signalling [163,179,180]. The gut microbiota mainly influences zinc bioavailability, uptake, and secretion [181]. Pregnant women are particularly vulnerable to zinc deficiency. Sauer and Grabrucker conducted a study on pregnant mice that were fed different diets for 8 weeks—with adequate zinc, zinc deficiency, or adequate zinc but high zinc uptake antagonists. The results showed that pregnant mice with acute zinc deficiency and pregnant mice on diets rich in zinc uptake antagonists had altered composition of gastro-intestinal microbiota. Changes in gastrointestinal permeability markers and signs of nervous system inflammation in the brain were noted. These effects were partially reversed and alleviated through supplementation with zinc–amino acid conjugates (ZnAA) [179]. In studies conducted on animal models, zinc deficiency has been shown to lead to changes in the composition of the intestinal microbiota [182,183,184]. For example, chronic zinc deficiency in chickens causes a reduction in gut microbial diversity and the growth of bacteria particularly adapted to low-zinc conditions, such as *Firmicutes*, *Prooteobacteria* and *Enterobacteriaceae*, leading to dysbiosis [185]. However, it should also be noted that excess zinc in the body can cause adverse effects. In a study conducted on mice infected with *Clostridium difficile*, excess zinc in the diet severely exacerbated the disease associated with this pathogen by altering the host immune response; increasing toxins; and expanding *Enterococcus*, *Porphorymonadaceae*, *Lachnospiraceae*, and *Clostridia cluster XI*, so zinc can also disrupt the balance of development of pathogenic and commensal strains [186]. A randomized double-blind placebo-controlled study by Surono et al. observed an increase in serum and faecal zinc levels in children who were given *Lactobacillus plantarum* probiotics and zinc at the same time [187]. Ballini et al. conducted a clinical study on a group of 40 patients between the ages of 14 and 18, divided into two categories (a treatment/active group and a placebo group), and showed that supplementation with a preparation containing the probiotics *Lactobacilllus plantarum*, *Lactobacillus acidophilus*, *Bifidobacteriuminfantis*, *Bifidobacterium lactis*, and prebiotic fructooligosaccharides (FOS) can help increase blood zinc levels, but the probiotics must be given for at least 5 weeks [188]. It has been shown that postbiotics and probiotics enriched with zinc and other minerals can exhibit anti-cancer, antioxidant and anti-inflammatory properties [162,189]. Mohammad Malyar et al., in a study conducted on Wistar rats, showed that supplementation with zinc-enriched probiotics can improve the growth performance of rats under heat stress due to antioxidant capacity; immune function; gene expression of Hsp90, Hsp70, SOD1, SOD2, MT1 and MT2; and changed morphological characteristics of villi height and intestinal wall thickness in the middle part of the jejunum [190].

Zinc is a cofactor for many enzymes involved in epigenetic regulation at the whole organism level. Transcriptional changes through histone acetylation and deacetylation are mediated by HDACs and histone acetyltransferases (HATs). Eighteen HDACs have been discovered and are divided into 4 classes—class I (HDAC 1–3 and 8), II (HDAC 4–7, 9 and 10), and IV (HDAC 11) require zinc for their enzymatic activity [171,191]. Zinc metalloenzymes are among the epigenetically active enzymes. Zinc is also essential for the self-regulation, catalysis, and integrity of such enzymes as DNA methyltransferase (DNMT), histone methyltransferase/methylase (HMT), histone demethylase (HDM), histone E3-ubiquitin ligase (EUBL), and histone deubiquitinating module (DUBm) complex. Among others, processes essential for DNA and histone methylation, such as methionine synthase and betaine-homocysteine methyltransferase, are zinc dependent. Also worth mentioning is that zinc finger proteins (ZFPs), many transcription factors, regulatory proteins, and other types of DNA-binding proteins contain zinc finger domain (ZFD), which is involved in epigenetic mechanisms [192,193]. Zinc interacts with the intestinal microbiota and can influence the course of intestinal diseases through a bidirectional relationship with the intestinal microbiota. Intracellular and extracellular zinc concentrations can affect the behaviour of the intestinal microbiota and vice versa, particularly when there is a change in the qualitative and quantitative composition of the intestinal microbiota. Zinc deficiencies are associated with increased intestinal permeability, while normal zinc levels, or supplementation in the case of deficiency, enhance the barrier function of the intestinal mucosa. Zinc is involved in the repair, anti-inflammatory, and modulating mechanisms of intestinal mucosal integrity [180,194,195]. In one study, zinc carnosine (ZnC) supplementation stabilized intestinal cell mucosa and reduced gastrointestinal damage by enhancing intestinal repair processes in rats and mice. The study showed that ZnC has biological activity, which was evaluated using several models of intestinal integrity and repair in a clinical trial [196]. In vitro studies in the CACO-2/T7 human intestinal cell line have shown that zinc deficiency can increase intestinal cell death, impairing intestinal permeability and tight junction integrity, through activation TNF-α. TNF-α promotes a zinc-dependent survival pathway that includes modulation of gene expression of transcription factors and signalling proteins [197]. Zinc is involved in DNA methylation, through which it has a major effect on the epigenome. Zinc deficiency during the prenatal period and childhood can contribute to altered promoter methylation, causing immune system dysregulation that can lead to the development of chronic inflammatory diseases. Because of the importance of zinc in the function of epigenetic enzymes, studies suggest that zinc deficiency may interfere with biological activities related to epigenetic mechanisms in offspring [198,199]. In their experiments, Li et al. investigated the effects of adequate or excessive maternal zinc intake on offspring intestinal immunity and basic epigenetic mechanisms in broiler chickens. They showed that maternal zinc supplementation during pregnancy is associated with lower levels of DNA methylation in intestinal cells, which in turn may have anti-inflammatory effects on the intestinal mucosa and increase intestinal mucosal barrier function through secretion of IgA (sIgA) and an increase in mucin 2 (MUC2). A20 is an anti-inflammatory protein that inhibits ubiquitin-dependent nuclear factor κB (NFκB) signalling regulated by zinc. A zinc-rich maternal diet alleviates intestinal inflammation through DNA hypomethylation and histone H3 at lysine 9 (H3K9) hyperacetylation in the A20 promoter of chick offspring [200. For a summary of the above section, see Table 2.

## 5. Conclusions

Current understanding suggests that nutrients and their terminal metabolites, such as SCFAs, can modulate the gut microbiota. Additionally, there is increasing evidence that microbes and their metabolites influence gene transcription within the gut, potentially increasing the risk of disease development. The relationship between epigenetic regulation and gut microbiome interactions appears to be bidirectional. This implies that alterations in the gut microbiota can induce epigenetic changes, while changes in epigenetics can also influence the composition and quantity of the gut microbiota. Understanding these relationships is essential to justifying and explaining how gut dysbiosis may contribute to the development of various diseases. On the other hand, the maintenance of a healthy gut microbiota composition may provide potential support for basic therapeutic strategies.

## Figures and Tables

**Table 1:** Links between gut microbiota and magnesium.

Intervention	Study Population	Results	References
**Research on animal models**			
A standard/control diet or a magnesium-deficient diet for 4 or 21 days (500 mg vs. 70 mg mg/kg).	Animal model—8 male C57Bl/6J mice (9 weeks old).	Magnesium-deficient diet reduced abundance of *Bifidobacterium*, which is associated with systemic inflammation and intestinal inflammation.	Pachikian et al. [156]
A standard/control diet or a magnesium deficient diet for 6 weeks (500 mg vs. 50 mg mg/kg).	Animal model—30 male C57BL/6NBomTac mice (8 weeks old).	Magnesium-deficient diet altered the composition of the gut microbiota and led to depressive-like behaviour.	Winther et al. [157]
Magnesium supplementation in colitis (30 mg vs. 1000 mg vs. 4000 mg mg/kg).	Animal model—27 female C57BL/6 mice (7–8 weeks old).	Increased abundance of *Bifidobacterium* and reduced abundance of pro-inflammatory *Enterobacteriaceae* alleviated colitis by modulating the gut microbiota.	Del Chierico et al. [158]
Dietary supplementation with a magnesium-rich marine mineral blend for 6 weeks (standard chow vs. 0.1% MMB-supplemented chow vs. 0.2%-supplemented MMB chow).	Animal model—30 male Sprague Dawley rats (7–8 weeks old).	A significant increase in the diversity of gut microbiota changed the profile of short-chain fatty acids (SCFAs) in the intestines compared to the control group.	Crowley et al. [160]
**Studies with humans**			
Probiotic *Lactobacillus reuteri DSM 17938* and magnesium oxide (MgO) for relieving chronic functional constipation in children (group A received *L. reuteri DSM 17938* and lactose hydrate as a placebo of MgO; group B received *L. reuteri DSM 17938* and MgO; group C received a placebo of *L. reuteri DSM 17938* and MgO).	60 children aged from 6 months to 6 years—a double-blind and randomized clinical trial.	*L. rueteri DSM 17938* combined with MgO was effective in treating functional constipation and did not disrupt the balance of the intestinal microbiota. MgO by itself suppressed the presence of the *Dialister* genus and disrupted the balance of the gut microbiota.	Kubota et al. [167]
Personalized magnesium supplementation for the prevention of colorectal cancer.	240 participants—a double-blind factorial randomized controlled trial.	Enhanced gut microbial production of medium-chain fatty acids (MCFAs) which consequently increased plasma MCFAs levels.	Fan et al. [168]

**Table 2:** Links between gut microbiota and zinc.

Intervention	Study Population or Materials	Results	References
Four groups of mice over 8 weeks (5 before and 3 during pregnancy): diet 1—a standard laboratory food (41 mg/kg zinc); diet 2—a zinc-deficient diet (19 mg/kg zinc); diet 3—a standard laboratory food (41 mg/kg zinc) with increased levels of phytates, folic acid, calcium, and iron; diet 4—diet 3 plus 41 mg/kg zinc–amino acid conjugate (ZnAA) supplement.	Animal model—female C57BL/6JRj mice (8 weeks old).	Zinc-deficient diet contributes to abnormal gut–brain signalling by altering intestinal physiology and the composition of the gut microbiota and increasing levels of anti-inflammatory cytokines. These effects were partially reversed and alleviated through supplementation with ZnAA.	Sauer and Grabrucker [179]
A standard/control diet 42 µg/g zinc and a zinc deficient diet 2.5 µg/g zinc administered to two groups of chicks.	Animal model—12 chicks *Gallus gallus* (upon hatching).	Chronic zinc deficiency alters gut microbiota composition and function and leads to increased abundance of *Firmicutes, Prooteobacteria,* and *Enterobacteriaceae*.	Reed et al. [185]
Zinc-enriched probiotics (ZnP). The rats (three groups) were fed a basal diet (control), basal diet with probiotics, or basal diet with zinc-enriched probiotic supplementation (ZnP, 100 mg/L) for 40 days under high heat stress.	Animal model—36 male *Wistar* rats (6 weeks old).	Rats showed improved growth performance under heat stress due to antioxidant capacity, immune function, expression genes, and change in morphological features of villi height and intestinal wall thickness in the middle part of the jejunum.	Malyar et al. [190]
A controlled zinc-deficient diet (20 mg/kg Zn), an adequate zinc diet (70 mg/kg Zn), or a zinc-supplemented diet (320 mg/kg Zn) for 6 weeks. After hatching, the offspring birds were fed diets with different zinc contents for 6 weeks as well.	Animal model—female chicks—broilers (45 weeks old) and their offspring.	Maternal high-zinc diet attenuates intestinal inflammation by reducing DNA methylation and elevating H3K9 acetylation in the A20 promoter of offspring chicks.	Li et al. [200]
The effect of zinc carnosine (ZnC) on various models of gut injury and repair in a clinical trial. Clinical trial: randomized crossover study comparing changes in intestinal permeability (lactulose/rhamnose ratio) before and after 5 days of treatment with indomethacin (50 mg three times a day) vs. ZnC (37.5 mg twice a day) or placebo.	Human colonic epithelial cells (HT29), rat intestinal epithelium (RIE) and canine kidney (MDCK). Rat model of gastric injury (indomethacin/restriction) and mouse model of small intestinal injury (indomethacin). Ten healthy volunteers (clinical trial).	ZnC stabilized intestinal cell mucosa and reduced gastrointestinal damage by enhancing intestinal repair processes in rats and mice. The study showed that ZnC has biological activity.	Mahmood et al. [196]
The effect of zinc on tumour necrosis factor alpha (TNF-α) triggered signalling in human intestinal cells.	A human intestinal cell line Caco-2/TC7 of clonal origin derived from high-passage parental Caco-2 cells.	Intracellular zinc is essential for maintaining intestinal epithelial integrity when cells are exposed to the inflammatory cytokine TNF-α. Zinc deficiency can increase intestinal cell death by impairing intestinal permeability and tight junction integrity through activation of TNF-α.	Ranaldi et al. [197]
